# In Vivo MR Imaging of Intraarterially Delivered Magnetically Labeled Mesenchymal Stem Cells in a Canine Stroke Model

**DOI:** 10.1371/journal.pone.0054963

**Published:** 2013-02-07

**Authors:** Shan-shan Lu, Sheng Liu, Qing-quan Zu, Xiao-quan Xu, Jing Yu, Jian-wei Wang, Yu Zhang, Hai-bin Shi

**Affiliations:** 1 Department of Radiology, The First Affiliated Hospital of Nanjing Medical University, Nanjing, Jiangsu Province, China; 2 State Key Laboratory of Bioelectronics, Jiangsu Key Laboratory for Biomaterials and Devices, School of Biological Science & Medical Engineering, Southeast University, Nanjing, Jiangsu Province, China; Stanford University School of Medicine, United States of America

## Abstract

**Background:**

This study aimed to evaluate the feasibility of intraarterial (IA) delivery and in vivo MR imaging of superparamagnetic iron oxide (SPIO)-labeled mesenchymal stem cells (MSCs) in a canine stroke model.

**Methodology:**

MSCs harvested from beagles’ bone marrow were labeled with home-synthesized SPIO. Adult beagle dogs (n = 12) were subjected to left proximal middle cerebral artery (MCA) occlusion by autologous thrombus, followed by two-hour left internal carotid artery (ICA) occlusion with 5 French vertebral catheter. One week later, dogs were classified as three groups before transplantation: group A: complete MCA recanalization, group B: incomplete MCA recanalization, group C: no MCA recanalization. 3×10^6^ labeled-MSCs were delivered through left ICA. Series in vivo MRI images were obtained before cell grafting, one and 24 hours after transplantation and weekly thereafter until four weeks. MRI findings were compared with histological studies at the time point of 24 hours and four weeks.

**Principal Findings:**

Home-synthesized SPIO was useful to label MSCs without cell viability compromise. MSCs scattered widely in the left cerebral hemisphere in group A, while fewer grafted cells were observed in group B and no cell was detected in group C at one hour after transplantation. A larger infarction on the day of cell transplantation was associated with more grafted cells in the brain. Grafted MSCs could be tracked effectively by MRI within four weeks and were found in peri-infarction area by Prussian blue staining.

**Conclusion:**

It is feasible of IA MSCs transplantation in a canine stroke model. Both the ipsilateral MCA condition and infarction volume before transplantation may affect the amount of grafted cells in target brain. In vivo MR imaging is useful for tracking IA delivered MSCs after SPIO labeling.

## Introduction

Stem cell based therapies have been shown to improve functional outcome in many experimental stroke. The likely mechanisms have been suggested as the neuroprotection [1]–[3], angiogenesis [4]–[6], axon-myelin remodeling [7], endogenous cell proliferation and possible replacement of damaged cells [8]. In the past years, because of relatively low immunogenicity and easy way of isolation, many studies used mesenchymal stem cells (MSCs) for stroke and suggested their effectiveness in cerebral ischemia. However, most of those studies were performed in rodents with intravenous or stereotactic injection of stem cells [4], [9], [10]. Unfortunately, the rodent brain is not gyrencephalic like the human brain. Besides, intravenously delivered cells pass through the systemic and pulmonary circulation systems which significantly reduce cells homing to the injured brain. Intracerebral injection is highly invasive and requires craniotomy [11]. Intuitively, the intra-arterial (IA) route of delivery, given that the fist pass of MSCs would be brain, seems better and enables widespread cerebral engraftment of cells. Furthermore, IA delivery of MSCs is clinically available because transfemoral catheterization of selected arteries has already been widely performed [12]–[14].

Magnetic resonance imaging (MRI) has been used to evaluate distribution and migration of stem cells after labeled with superparamagnetic iron oxide (SPIO) contrast agents [15]–[17]. To our knowledge, few studies have addressed in vivo MRI tracking of intra-arterially delivered MSCs in large animals like beagle dogs which are structurally similar to the human brain.

The aims of the present study were as follows: 1. Is it feasible of MSCs transplantation by IA route in an experimental canine model of cerebral infarction? 2. Can 3.0 Tesla (T) MRI effectively track the IA transplanted SPIO-labeled MSCs?

## Materials and Methods

### Synthesis of Fe_3_O_4_-DMSA-PLL (SPIO)

0.5 mol·L^−1^FeCl_3_ and 0.25 mol·L^−1^ FeSO_4_·7H_2_O were dissolved in 100 ml deionized water in a three-neck flask followed by adding 2 mol·L^−1^ NH_3_·H_2_O solution with stirring at 70°C under a N_2_ atmosphere. Then NH_3_·H_2_O was progressively added to adjust the aqueous dispersion pH to 11.0 - 12.0. The temperature was raised to 65°C and the system was then mixed for two hours at 65°C. 30 ml oleic acid (OA) was slowly added to the solution. The pH was adjusted with dilute hydrochloric acid (HCL) solution to 6.0 - 7.0, and the temperature was raised to 80°C for one hour, and then cooled down to room temperature with the pH controlled at 3.4 - 4.0. Black precipitates were collected by filtration and carefully washed with deionized water and ethanol several times. The obtained Fe_3_O_4_-OA nanoparticles were dissolved in 100 ml hexanol.

0.65 mmol Fe_3_O_4_-OA and 0.3 mmol meso-2,3-Dimercaptosuccinic acid (DMSA) were dissolved in 50 ml acetone with stirring at 60°C for four hours. Then Fe_3_O_4_-DMSA nanoparticles were collected by magnetic separation, washed with ethanol and deionized water several times and dissolved in 20 ml deionized water.

To 20 ml of 0.5 mg/ml poly-l-lysine (PLL) solution, 20 ml of 0.175 mg/ml Fe_3_O_4_-DMSA were added under stirring. The mixture was sonicated for 20 minutes and then further stirred for two hours. The Fe_3_O_4_-DMSA-PLL nanoparticles were obtained by removing free PLL via ultrafiltration.

### Cell Culture and Labeling

Bone marrow was obtained from the humerus of dog two weeks before embolization. Autologous MSCs were isolated and purified by density gradient centrifugation and adhering to the wall. Briefly speaking, the bone marrow was diluted (1∶1) in phosphate buffered saline (PBS), layered onto a density gradient solution (Ficoll-Paque, TBDscience, Tianjin, China) and centrifuged for 25 minutes at 300 grams. After that, enriched cells were removed from plasma-solution interphase, washed twice with PBS, and cultured in low-glucose Dulbecco’s modified Eagle’s medium (DMEM, Gibco, Karlsruhe, Germany) supplemented with 0.2 mmol/ml of L-glutamine, 100 U of penicillin, 100 g/ml of streptomycin and 10% fetal bovine serum (FBS, Gibco, Karlsruhe, Germany) at 37°C and 5% CO_2_ [18]. Three days later, the culture solution was replaced for the first time, and nonadherent cells were removed at the same time. The medium was changed every three days. After the cultures reached 90% confluence, the cells were harvested with 0.25% trypsin (Gibco, Karlsruhe, Germany) and replated.

Passage three cells were co-cultured in fresh medium containing various concentrations of SPIO (5, 10, 20, 40, 80 µg/ml) for 24 hours according to the previous reports [19] and then were washed three times in PBS to eliminate extracellular SPIO.

After that, prussian blue (PB) staining was performed to show intracellular iron. The cells were incubated with 2% potassium ferrocyanide in 6% HCL for 15 minutes and then counterstained with nuclear fast red for one minute. The intracellular iron content was also quantified before and after cell labeling by atomic absorption spectrometry (Shengyang, China) as described previously [19]. The average iron content per cell was calculated according to the cell numbers.

### Cellular Viability and Apoptosis

For assessment of cell viability, cultures were incubated with 0.4% trypan blue (Sigma, St. Louis, MO, USA). Cell viability was determined according to the percentage of cells not internalizing the dye.

For assessment of cell apoptosis, labeled cells (1×10^6^) and unlabeled cells (1×10^6^) were collected, washed twice with cool PBS and resuspended in 50 µl annexin medium, followed by adding 5 µl annexin V-fluorescein isothiocyanate (AV-FITC). The suspension was then kept in dark for 15 minutes at room temperature. 10 µl propichium iodide (PI) was added and the suspension was again stored in dark for five minutes. After that, the cells were washed twice again with cool PBS and resuspended with 300 µl annexin. Flow cytometer (BD FACSCalibur, CA, USA) was used to determine the percentage of cells undergoing early apoptosis (annexin^+^/PI^-^) and late apoptosis or death (annexin^+^/PI^+^).

### Animal Ischemia Model Establishment and the Transplantation Procedure

All the experimental procedures were approved by the local Animal Experiment Ethical Committee. 14 beagle dogs (body weight, 13.9±1.0 kg) were enrolled and randomized to transplantation group (n = 12) and control group (n = 2).They were anesthetized with 3 mg/kg pentobarbital intravenously during all the procedure. Rectal temperature was continuously monitored and maintained at 37°C to 39°C.

The left proximal MCA was embolized with autologous thrombus (1.7 mm in diameter and 5 mm in length) prepared approximately two hours before as described in our previous study [20]. Under angiography (Axiom Artis; Siemens AG, Muenchen, Germany), a 5 French vertebral catheter (Terumo Medical Corporation, Somerset, NJ, USA) was advanced 2 cm into the ascending part of left internal carotid artery (ICA). One thrombus was injected into the ICA with 3 ml of 0.9% saline. Complete left proximal MCA occlusion (MCAO) was verified by following angiography. Then the catheter was kept in the left ICA for two hours for continuously occluding the blood flow. All the dogs were kept warm with heating blanket and observed continuously until they had fully recovered from anesthesia. Intramuscular ampicillin (20 mg/kg) was given to all of them daily for up to one week.

One week after ischemia, all the animals in experimental group underwent angiography before MSCs transplantation. They were then classified as three groups according to the angiography findings. Group A: dogs with complete left MCA recanalization and distal blood flow; group B: dogs with incomplete left MCA recanalization regardless whether there was distal blood flow or not; group C: dogs with persistent left proximal MCA occlusion without any distal blood flow. A bolus of cell suspension with about 3×10^6^ MSCs inside was slowly injected over a five-minute period into all the dogs using a 10 ml syringe through a 5 F vertebral catheter in the left ICA, followed by 5 ml 0.9% saline. The catheter was left in place for 10 minutes before withdrawal to minimize cell backflow.

### In vivo MR Imaging

3.0 T MRI scanner (Magnetom Trio, Siemens Medical Systems, Germany) and an eight-channel knee coil were used in the study. Dogs were positioned supinely in the MR scanner with head in the center of the coil.

T2 weighted imaging (T2WI) and diffusion weighted imaging (DWI) were performed at six hours, 24 hours and one week after MCA embolization and temporal ICA occlusion. T2^*^ weighted imaging (T2^*^WI), T2^*^ map and susceptibility weighted imaging (SWI) were added to in vivo MRI imaging and were obtained just before cell grafting and at one hour, 24 hours and weekly thereafter until four weeks after IA transplantation. The parameters were as follows: T2WI: TR 5000 ms, TE 60 ms, matrix 320×320; T2^*^WI: TR 1460 ms, six TEs (20, 25.2, 30.4, 35.4, 40.8, 46.0 ms), matrix 320×320; T2^*^ map: TR 80 ms, TE 20 ms, matrix 256×256; SWI: TR 28 ms, TE 20 ms, matrix 448×448, and DWI: TR 5500 ms, TE 96 ms, matrix 192×192, b value 0 and 1000 sec/mm^2^. Other parameters were the same: FOV 200 mm, 22 slices, and slice thickness 2.0 mm with no gap.

### MRI Data Processing

One experienced radiologist (XQ Xu) measured visible ischemic lesions by drawing regions of interest around the lesions on T2WI images at 24 hours and one week after embolization. Image brightness and contrast were optimized between areas of abnormal tissue and normal appearing brain. Lesion volumes were obtained by multiplying the lesion areas by the section and gap thickness.

T2^*^ value of transplantation group was measured from T2^*^ maps by one radiologist (QQ Zu). The ROIs were selected to avoid susceptibility effects arising from air tissue interface and hemorrhage. Each ROI area was 20 mm^2^. ROIs were placed on the peri-infarction (PI) area, ipsilateral normal parenchyma (INP) and infarction itself, according to the corresponding T2WI and SWI, respectively. T2^*^ ratios were got after normalizing with the contralateral mirror region. Due to the heterogeneity of T2* maps, we measured T2* ratio of each area three times in each dog. The mean value of T2* ratio was used for further analyses.

### Histopathological Analysis

Two dogs (dog 8, 10) were sacrificed at 24 hours after transplantation to localize the MSCs. Other dogs were sacrificed at the fourth week. The brains were removed and immersed in 4% paraformaldehyde in PBS at 4°C for two days and then cut into 2-mm thick coronal blocks, followed by embedded in paraffin. Four µm thick coronal sections were sliced from each block and stained for hematoxylin and eosin (HE staining).

To visualize the transplanted MSCs in the host brain, sections were processed for PB staining. The sections were incubated for 30 minutes with 2% potassium ferrocyanide in 6% HCL, counterstained with eosin and viewed under light microscope (model AH3, Olympus, Tokyo, Japan).

### Neurological Scoring

Neurological scoring was performed using a standardized categorical rating scale according to previous report [21]. It was consisted of evaluation for motor function (no deficit = 1, hemiparetic but able to walk = 2, stands only with assistance = 3, hemiplegic and unable to stand = 4, comatose or dead, not testable = 4), consciousness (normal = 1, mildly reduced = 2, severely reduced = 3, comatose or dead = 4), head turning (absent = 0, posturing and turns toward side of infarct = 1, unable to lift head, comatose, or dead = 1), circling (absent = 0, present = 1, does not walk or dead = 1) and hemianopsia (absent = 0, present = 1, unable to test because of reduced consciousness or death = 1). Dogs were evaluated at 24 hours, one week after ischemia and then weekly until sacrifice.

### Statistical Analysis

All data were reported as mean ± standard deviation (SD). The student *t* test was used to determine whether there was difference between SPIO-labeled MSCs and control group. One-way analysis of variance followed by a post-hoc test was applied to assess the T2^*^ ratio differences among the three groups and between any two groups one hour after IA transplantation. The *P*-value was two-sided and value of less than 0.05 was considered statistically significant. All analyses were performed using SPSS (Version 13.0, Chicago, Illinois).

## Results

### Fe_3_O_4_-DMSA-PLL Nanoparticles

**Figure 1:**
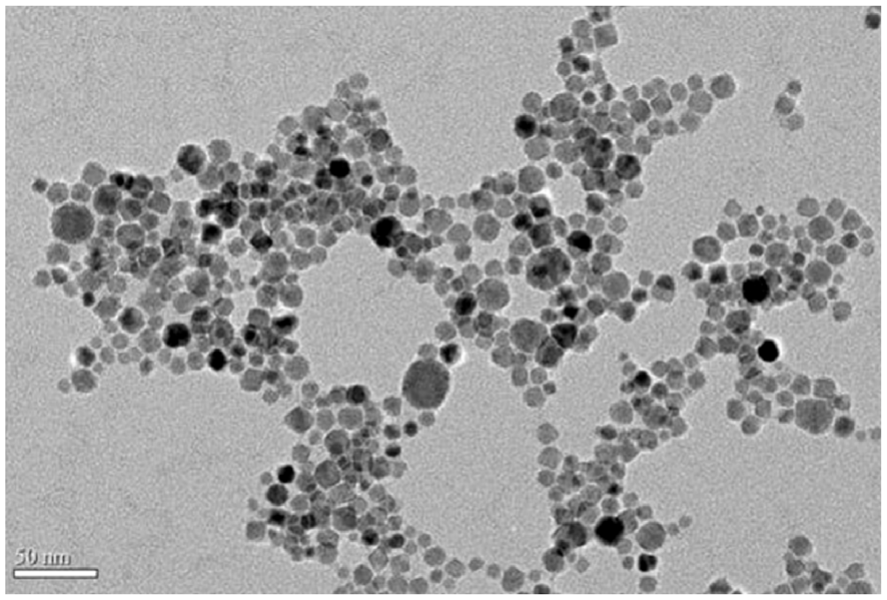
The transmission electronic microscopy image of home-synthesized Fe_3_O_4_ nanoparticles. The average size of Fe_3_O_4_ nanoparticles calculated from 100 particles at least was 12 nm.

The prepared Fe_3_O_4_-DMSA-PLL nanoparticles were stable and fully water-soluble. The transmission electronic microscopy (TEM, JEOL JEM-2100) image showed the Fe_3_O_4_ nanoparticles with the average size of 12.2±4.3 nm (Figure 1). Hydrodynamic size and zeta potential of Fe_3_O_4_-DMSA-PLL nanoparticles were 152.8±2.2 nm and +43.43±6.1 mV.

### Cell Culture, Labeling, Viability and Apoptosis

**Figure 2:**
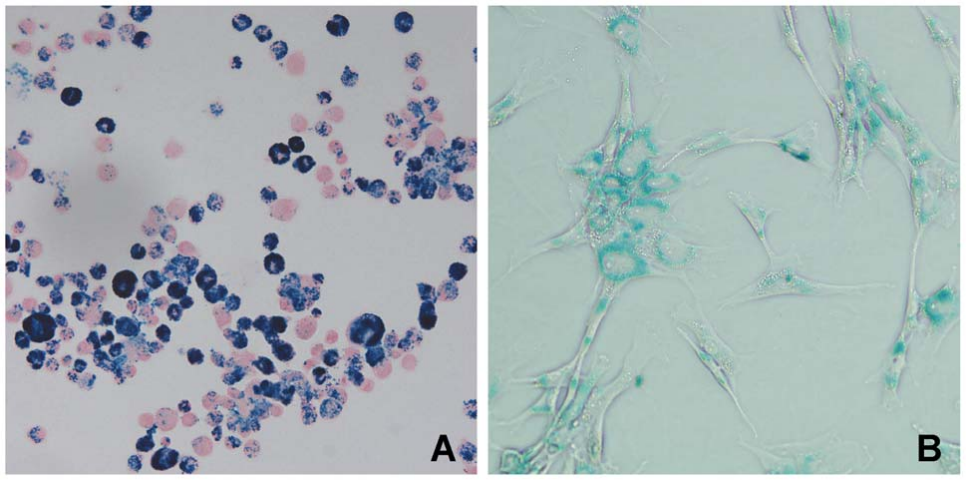
Photomicrography of MSCs after incubated with Fe_3_O_4_-DMSA-PLL. Prussian blue stained smear of labeled MSCs showed most of the cells were prussian blue positive (A). Intracytoplasmic blue particles were clearly visible under inverted phase contrast microscope (B).

After PB staining, blue particles were seen in almost every MSC (Figure 2A–B). As the concentration of SPIO increased from 5–80 µg/ml, the blue cytoplasmic inclusions increased proportionally. Low concentration (20 µg/ml) was favored for further use considering of safety. The mean intracellular iron was 33.14±4.35 pg/cell in labeled cells in comparison with 1.56±0.47 pg/cell in the unlabeled cells.

**Figure 3:**
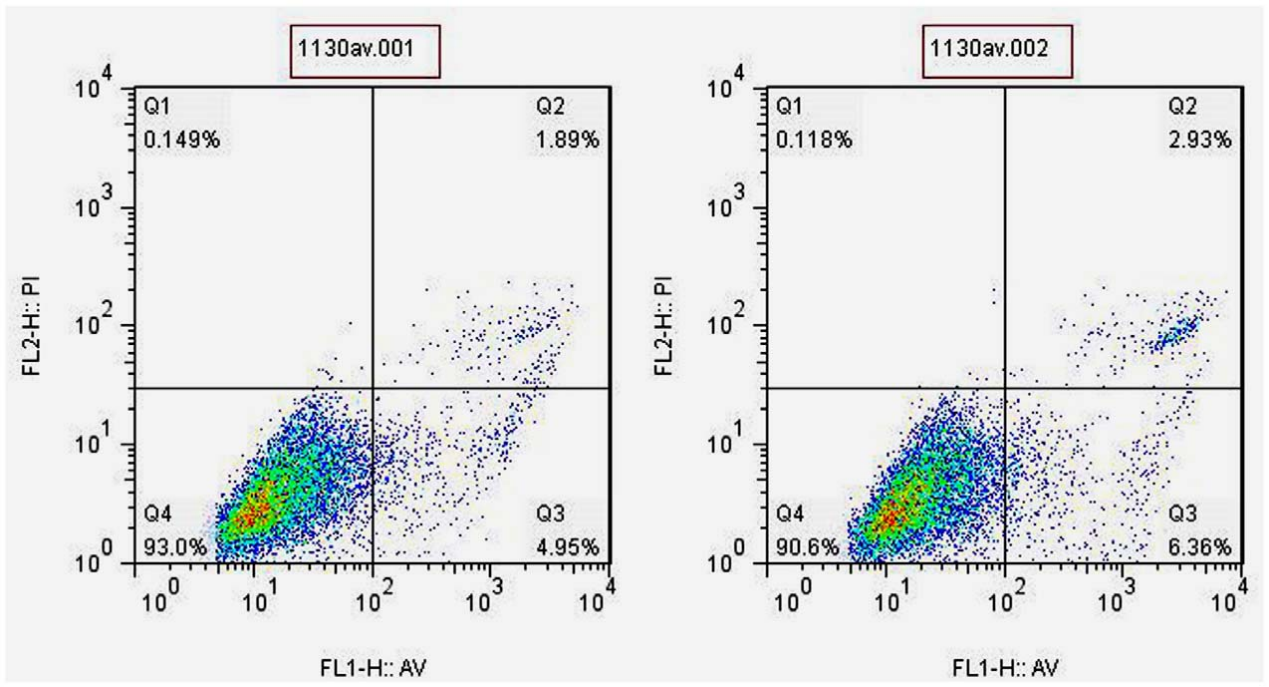
AV-FITC-PI double staining for MSCs apoptosis after labeling. Early apoptotic percent (lower right quadrant) were 4.95% and 6.36% while late apoptotic and necrotic percent (upper right quadrant) were 1.89% and 2.93%, respectively in unlabeled cells (left) and labeled cells (right). The cross inside was gate.

Trypan blue staining showed SPIO loading had no effect on cell viability when compared with control group (*t* = 1.682, *P*>0.05). The mean viability was 94.71% ±1.48% and 96.14% ±1.46%, respectively. Cell apoptosis test showed that total apoptosis and necrosis rate were 8.86% ±3.56% and 7.66% ±1.31% in the labeled and control group, respectively (Figure 3). There was no statistical differences between them (*t = *0.71, *P*>0.05).

### Establishment of Cerebral Ischemia

**Figure 4:**
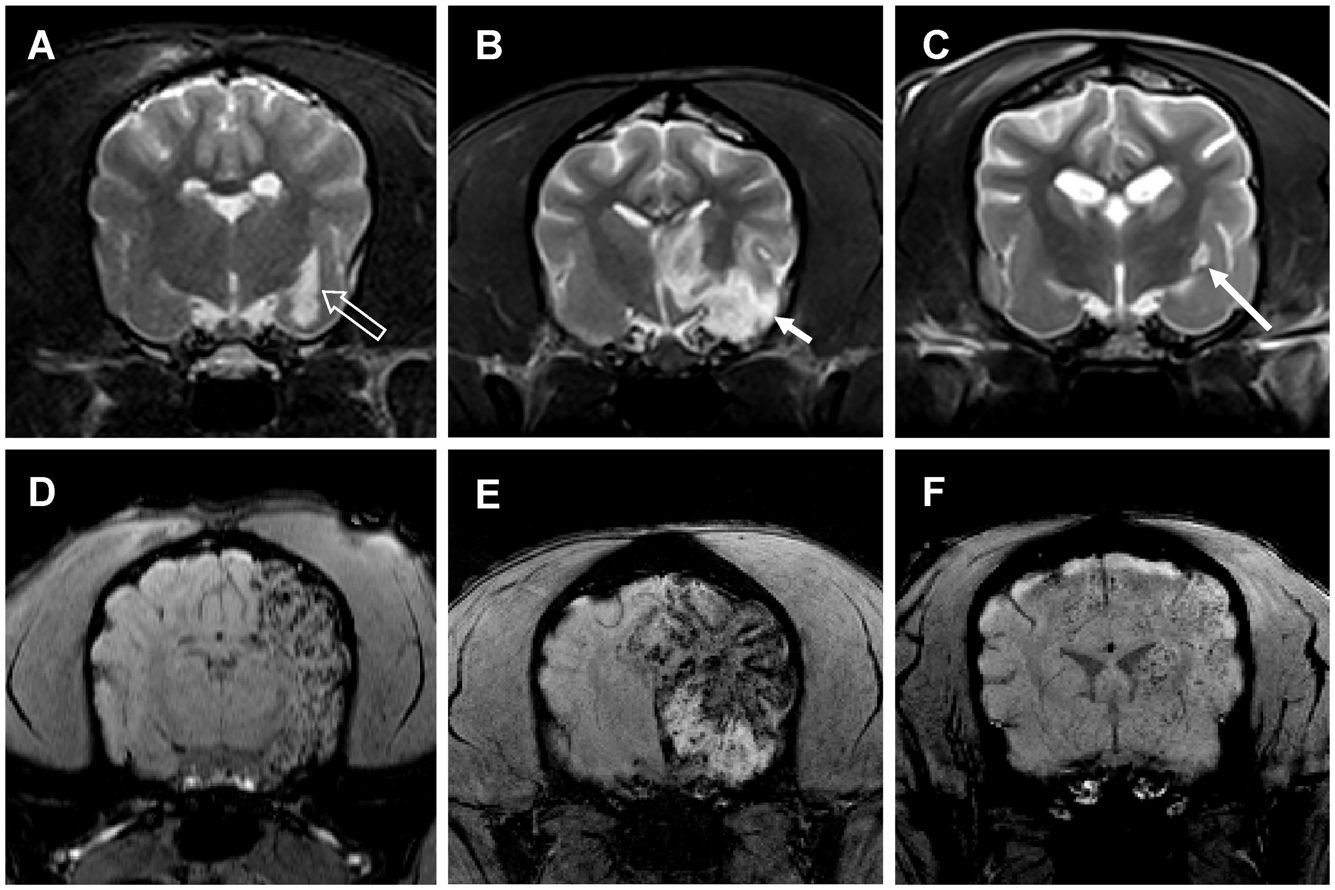
Three types of ischemic infarction and the amount of grafted MSCs in the target brain. Moderate size of cerebral infarction involving the left temporal lobe (A), large infarction in left MCA territory (B) and small infarction located in subcortical white matter (C) were got after combining left proximal MCA embolization with two-hour left ICA blood flow occluding. All these three dogs (dog 5, 12 and 13) had complete left MCA recanalization before transplantation (not shown). After intra-carotid MSCs transplantation, dog with large infarction had more obvious MSCs in the brain than that with moderate size of infarction (D-E), while dog with very small infarction was shown fewer scattered cells (F).

After combining left proximal MCAO with two-hour left ICA blood flow occluding, moderate size of cerebral infarction, involving left basal ganglia and left temporal lobe including cortex and subcortical white matter, was observed in 10 dogs (Figure 4A). The mean volume on T2WI was 599.45±262.50 mm^3^ at 24 hours and decreased to 390.93±92.82 mm^3^ at one week (before transplantation). Three dogs (dog 4, 7, 12) had large MCA territory infarction before transplantation, respectively (Figure 4B). One dog (dog 13) only had small infarction involving the subcortical white matter of left temporal lobe, with the volume of 61.14 mm^3^ (Figure 4C).

One week after ischemia, there were five, four and three dogs distributed to group A, B and C, respectively, according to the angiography findings before MSCs transplantation in experimental group. The infarction volume on T2WI at 24 hours and one week after embolization and the details of dogs grouping before transplantation were summarized in table 1.

**Table 1:** Dog grouping and infarction volume on T2WI after embolization.

Grouping *	Dog ID	Infarct volume on T2WI after embolization (mm^3^)	
		24 hours	1 week
Group A	Dog 2	841.8	410.54
	Dog 5	819.94	319.74
	Dog 8	857.9	430.26
	Dog 12	3777.9	3185.16
	Dog 13	64.84	51.14
Group B	Dog 1	725.6	587.28
	Dog 9	468.36	319.36
	Dog 10	876.56	478.12
	Dog 3	230.26	304.1
Group C	Dog 14	251.36	423.64
	Dog 7	5902	3367.96
	Dog 11	318.52	312.52
Control group	Dog 4	3255.32	1960.9
	Dog 6	604.8	323.76

Note: * Group A: complete left MCA recanalization with distal blood flow before transplantation; Group B: incomplete left MCA recanalization regardless whether there was distal blood flow or not before transplantation; Group C: persistent left MCA occlusion without any distal blood flow before transplantation; Control group: dogs without cell transplantation.

### In vivo MRI Detection of Transplanted MSCs

**Figure 5:**
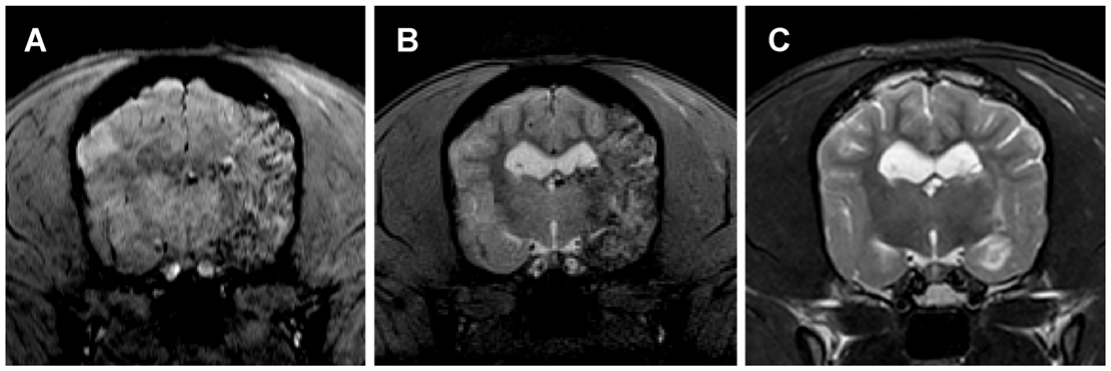
Comparison of three sequences for detecting IA transplanted MSCs. Grafted MSCs were shown obvious hypointensity and diffuse distribution on both SWI (A) and T2^*^WI images (B). T2WI image allowed a proper visualization of the ischemic brain tissue but it failed to detect the MSCs in the brain (C).

Both T2^*^WI and SWI could detect the MSCs in vivo clearly (Figure 5A–B). On T2^*^WI, the grafted cells were shown more obvious signal intensity loss along with TE time increasing from 20 ms to 46 ms while the signal-to-noise ratio of images decreased at the same time. T2WI allowed a proper visualization of the ischemic brain tissue but it failed to detect the MSCs in all the dogs (Figure 5C).

**Figure 6:**
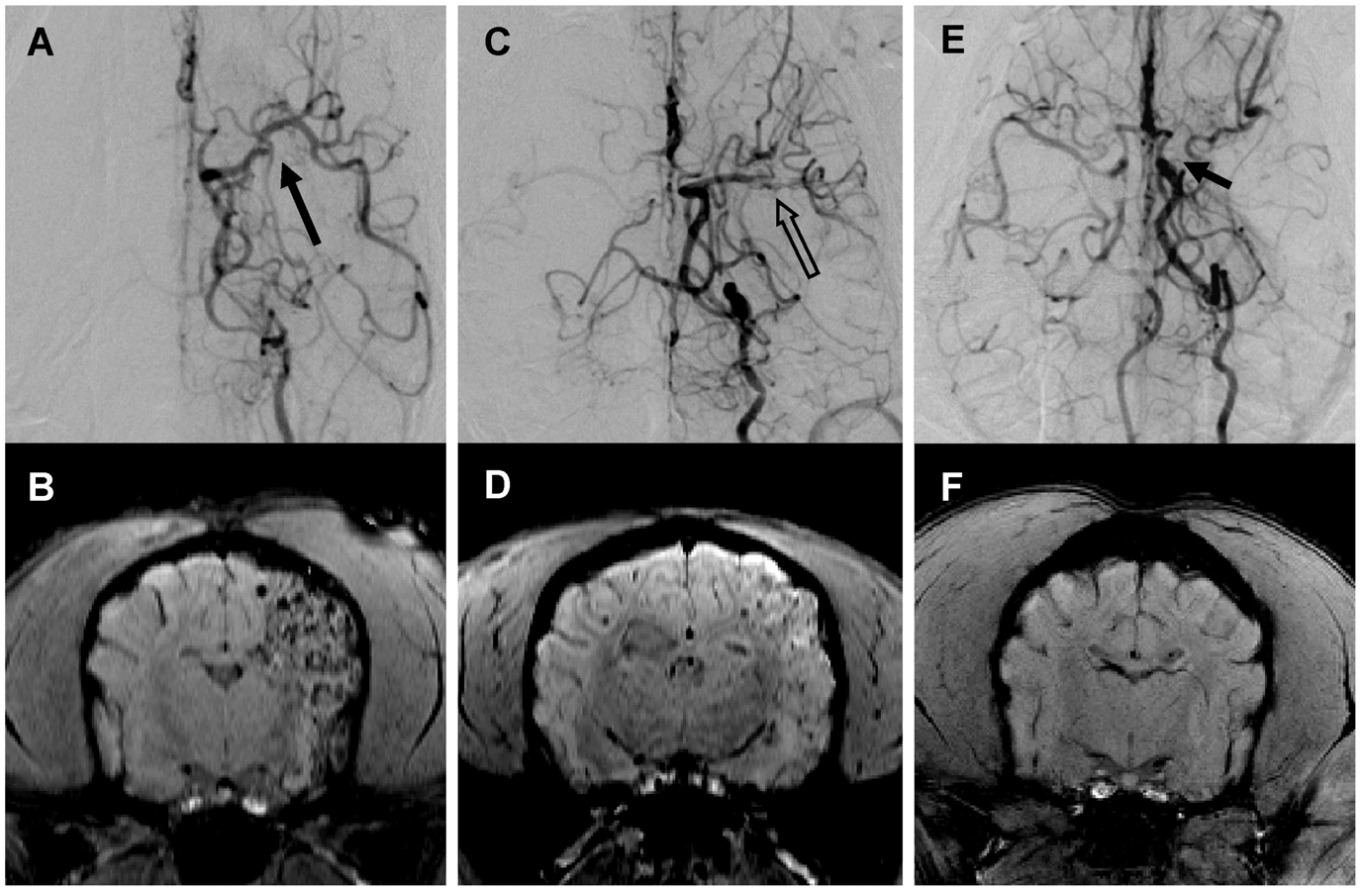
The amount of engrafted MSCs in the brain in three groups. (A-B) Large amounts of transplanted MSCs scattered in a wide range in the left cerebral hemisphere after IA delivery in group A with complete MCA recanalization (long arrow); (C-D) Fewer amounts of MSCs in the brain were observed in group B with incomplete MCA recanalization (empty arrow); (E-F) No definite hypointensity of MSC was observed in group C with continuous MCA occlusion before transplantation (short arrow).

In group A, large amounts of MSCs scattered discretely as small clusters in a wide range in the left cerebral hemisphere in four dogs one hour after IA administration (Figure 6A–B), except dog 13 which had very small infarction and was shown fewer scattered cell clusters (Figure 4F). Moreover, dog 12 with large infarction had more grafted MSCs than those with moderate size of infarction (Figure 4D–E). In group B, dogs were shown fewer amounts of MSCs in the brain by visual evaluation (Figure 6C–D). Grafted MSCs of two dogs (dog 3, 10) were mainly observed in the left basal ganglia while only small amount of cells were detected in the INP area. In group C, no visible MSC was observed by in vivo MRI imaging (Figure 6E–F).

The corresponding T2^*^ ratios of infarction, the PI and INP area in transplantation groups at one hour after transplantation were shown in table 2. There was significant difference of PI T2^*^ ratios among the three groups and between any two groups (*F* = 17.71, *P*<0.01). The significant differences of INP T2^*^ ratios were found between group A and B as well as group A and C (*F* = 10.54, *P*<0.01).

**Table 2:** T2^*^ ratios of infarction, PI and INP one hour after transplantation.

Area	T2^*^ ratio			*P*-value ^#^
	Group A	Group B	Group C	
PI	0.45±0.19	0.78±0.07	1.02±0.03	0.001
INP	0.46±0.23	0.82±0.15	1.02±0.04	0.004
Infarction	1.04±0.18	1.11±0.15	1.06±0.09	0.777

Note: PI: peri-infarction area, INP: ipsilateral normal parenchyma. # One-way analysis of variance was performed for the three groups. Data was reported as mean ± standard deviation.

**Figure 7:**
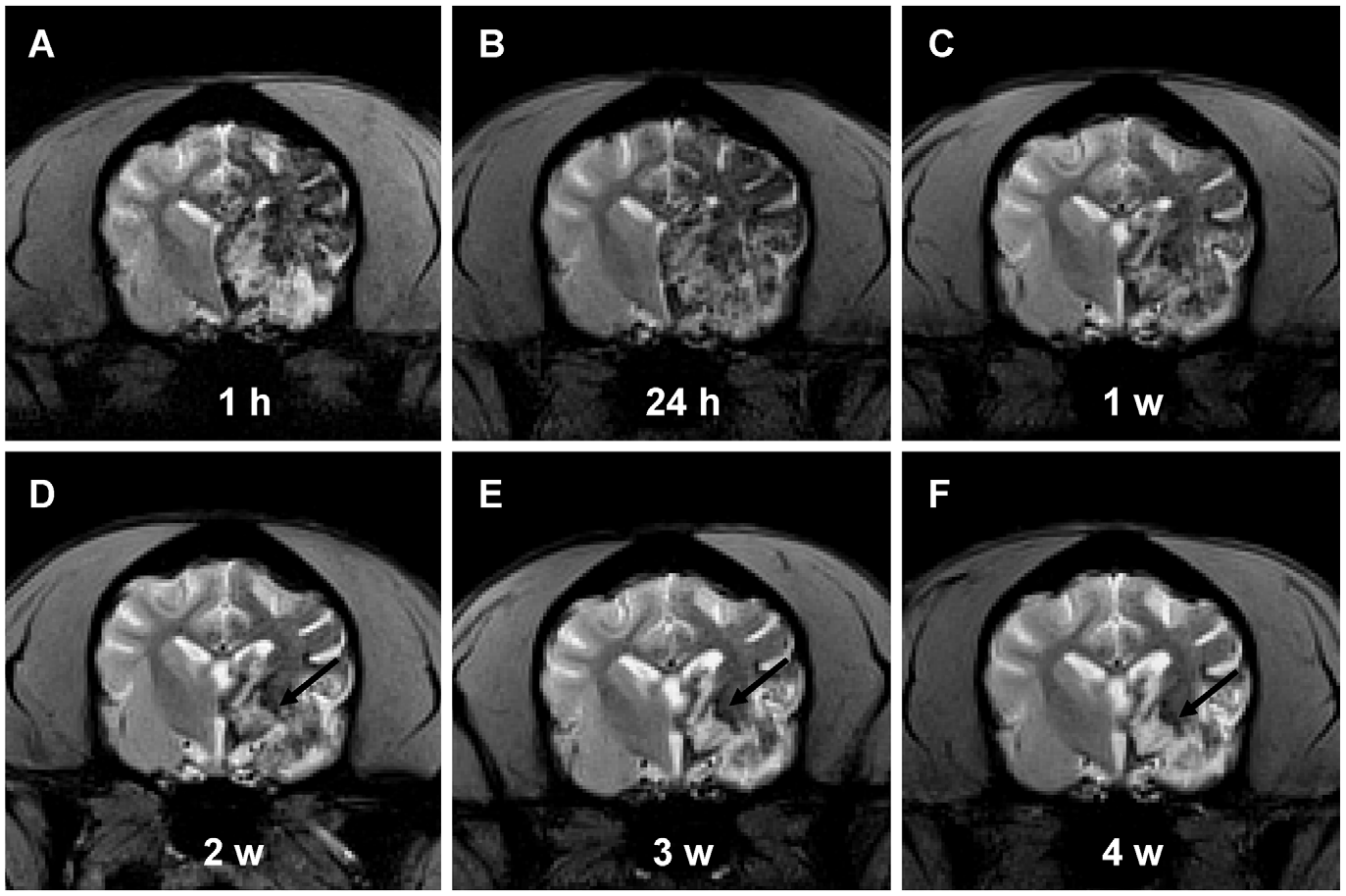
Serial T2^*^WI images from one hour to four weeks after cell transplantation. Hypointensity of MSCs generally kept decreasing but was still visible within four weeks (A-F). Hypointensity of the PI area (black arrow in D-F) showed a trend of slower decline than that of the INP area.

During the series tracking in four weeks, the hypointensity of MSCs generally kept decreasing but was still visible, accompanied with T2^*^ ratio increasing. The hypointensity of the PI area showed a trend of slower decline than that of the INP area (Figure 7A–F).

Two dogs (dog 2, 12) developed new cerebral infarction on DWI within 24 hours after transplantation. The volume was 549.68 mm^3^ (dog 2) and 358.08 mm^3^ (dog 12) respectively. Both of the two dogs had high cell engraftments in the brain. The new infarction appeared around the area of cell clusters. Another dog (dog 10) was suffered from epilepsy attack after recovery from anesthesia although the amount of grafted cells in the brain was low. It was sacrificed at 24 hours after transplantation.

### Neurological Scoring and Histological Findings

Twenty-four hours after the onset of ischemia, eight animals had mildly reduced consciousness and right side hemiparetic but able to walk, while three dogs (dog 4, 7, 12) had severely reduced consciousness and were unable to stand. Three dogs didn’t show any motor function deficit but had mildly reduced responsiveness. The circling movements, head turning and hemianopsia were observed in 11, 8 and 8 dogs, respectively. One week later, these signs were improved. Continuous improvement was observed within four weeks after MSCs transplantation. The mean neurological scorings before and after transplantation were summarized in Table 3.

**Table 3:** Neurological evaluation before and after MSCs transplantation.

Grouping	Before transplantation *		After transplantation			
	24 hours	1 week	1 week	2 weeks	3 weeks	4 weeks
Group A	6.20±2.17	4.60±1.52	4.00±1.83	3.50±1.29	3.00±1.15	2.50±0.58
Group B	5.75±0.96	4.50±1.29	3.33±0.58	2.67±0.58	2.33±0.58	2.33±0.58
Group C	6.67±2.89	5.33±2.52	4.00±2.00	3.67±2.08	3.33±1.53	3.00±1.00
Control group	7.50±2.12	6.50±2.12	5.50±2.12	4.50±2.12	4.50±2.12	3.50±0.71

Note: * After completing the procedure of left MCA embolization and temporal ICA occlusion. Data was reported as mean ± standard deviation.

**Figure 8:**
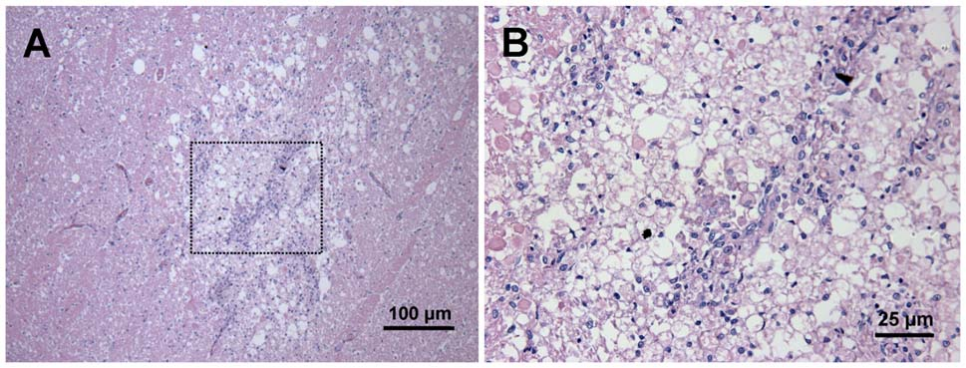
HE staining after infarction. (A) White matter involvement was observed after infarction (×100). (B) Prominent neuronal cell loss, necrotic neurons and nuclear karyolysis were shown, accompanied with aggregated macrophages and small amount of lymphocytes (dotted box in A, ×400).

**Figure 9:**
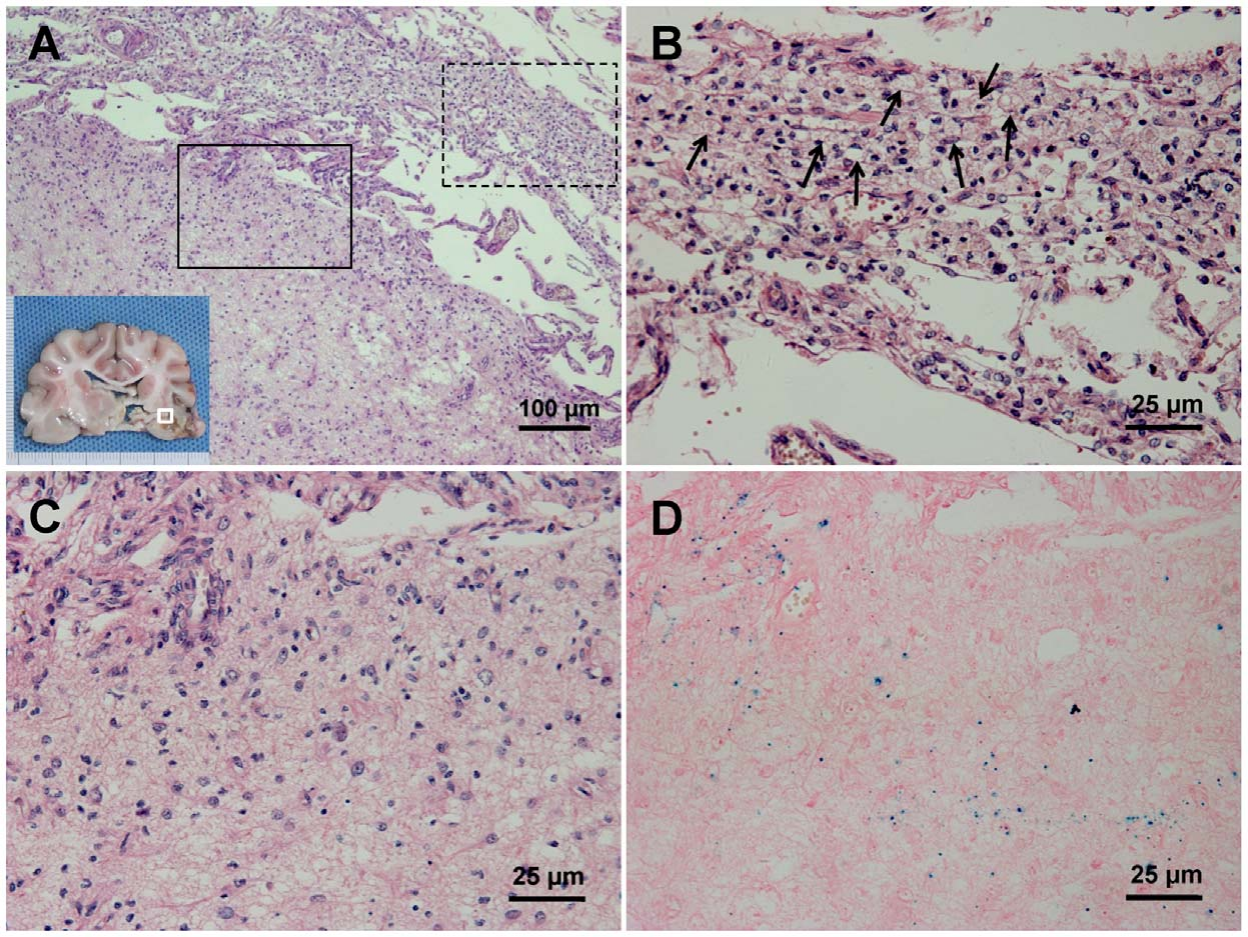
Histological findings at four weeks after MSCs transplantation. (A) Microscopic view of specimen (white box in the gross image) showed clear boundary between infarction and peri-infarction area (HE staining ×100). (B) Neuronal cell necrosis, tissue loss and aggravated macrophages (black arrows) were observed inside of infarction (dotted box in A, HE staining ×400). (C) No obvious macrophage was observed in the peri-infarction area (solid line box in A, HE staining ×400). (D) PB staining showed congregate PB-positive cells nearby lesion boundary (PB staining ×400).

**Figure 10:**
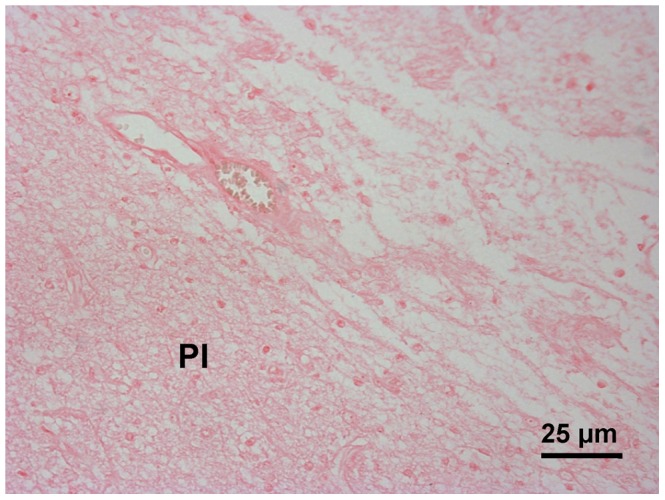
PB staining of the control group. No PB-positive cell was observed in the peri-infarction area (PI).

By HE staining, infarction involving the white matter was observed. Prominent neuronal cell loss and nuclear karyolysis were shown (Figure 8). PB stain-positive cells were observed mainly around the vessels at 24 hours after transplantation. Congregate PB-positive cells were observed nearby infarction boundary four weeks after transplantation (Figure 9A, C, D). Macrophages gathered inside of the infarction rather than the PI area (Figure 9B, C). Some scattered PB-positive cells were also found in brain parenchyma in dogs of group C although they were not detected by MRI in vivo. PB stain of PI area was negative in the control group (Figure 10).

## Discussion

Intra-arterially delivered MSCs was non-invasively and dynamically investigated by 3.0 T MRI after labeling with Fe_3_O_4_-DMSA-PLL in the present study. We showed that MRI could visualize the cells as soon as one hour after IA injection and was effective for tracking grafted MSCs within four weeks.

We choose beagle dog for IA transplantation because it is structurally similar to the human brain, has little variation in the size of the brain specimens and in the diameters of intracranial arteries despite the range of weight and technically easy for transfemoral catheterization of carotid artery [20], [22]. In our previous study [20], an embolic stroke model resembling lacunar infarction was got by proximal MCA occlusion in beagle dogs. In order to enlarge the cerebral infarction, proximal MCA occlusion in combination with temporal occlusion of the ipsilateral ICA which had been reported previously was used in our current study [23], [24]. Collateral supply was restricted by temporary ICA occlusion. Cerebral blood flow in the MCA territory was further reduced into the ischemic range which gave rise to the increase of infarction volume compared with single-thrombus approach. However, due to the variability of collateral circulation in dogs, heterogeneous infarction was observed in our study.

Results of labeling MSCs in vitro with home-synthesized SPIO demonstrated its usefulness to label MSCs without cell viability compromise which was compatible with widely used ferumoxides-PLL complex [25], [26]. Moreover, such labeled MSCs could be detected in vivo efficiently after IA transplantation by 3.0 T MRI. In agreement with previous studies [15], [27], [28], IA administration could produce a large number of MSCs in the target brain. The injected cells mainly distributed in the ipsilateral cerebral hemisphere of infarction while only few MSCs were found in the contralateral side.

However, no MSC was shown in dogs with occluded MCA at the time of transplantation. We considered two reasons for this. First, the main approach for MSCs entering brain may be the ipsilateral MCA. Thus, the flow status of that MCA before transplantation may play an important role in distribution and the amount of cells in the target tissue. Fewer MSCs detected in group B than group A could also confirm our speculation. Second, we considered that there should be a small amount of MSCs in the brain which was delivered to the host brain through other access, like anterior cerebral artery, as confirmed by PB staining in group C. However, our imaging methods may not be sensitive enough to detect small amount cells or tiny cell clusters.

In the present study, labeled MSCs were transplanted at one week after onset of ischemia. Strbian et al considered the blood-brain barrier (BBB) was continuously open for several weeks after focal cerebral ischemia [29]. Komatsu et al also found that BBB may largely break for at least two weeks after cerebral infarction and remain insufficient even four weeks [1]. Thus, we think that deficit BBB after cerebral ischemia may account for the mechanical trapping of MSCs from vessel to brain parenchyma as well as the possible cells immigration after transplantation.

The gradually fading of low signal intensity in the host brain was not only due to the cell division but also biodegradation and entry of iron into metabolic pathways [25], [30]. Even so, SPIO-labeled MSCs will retain the iron nanoparticles to a sufficient degree to produce hypointensity within four weeks on SWI and T2^*^WI. T2^*^ ratio could reflect the amount of grafted cells in the brain. But it is still difficult to directly extrapolate the exact number due to heterogeneity in the structure and composition of brain tissue and differences in MRI sequence and parameters [31].

The slower fading of hypointensity in PI area than in INP may support the concept that chemoattractive factors may be released in the site of brain lesion which gave rise to reservation and specific homing of transplanted cells to the PI area [32], [33]. PB staining confirmed that there were MSCs in the PI area.

Our results also showed that a smaller infarction on the day of cell transplantation seemed to be associated with fewer cells in the brain after IA delivery while dog with large infarction had more engrafted cells. But due to the small number of dogs, we didn’t perform statistical analyze. Li et al reported animal with smaller lesions (less than 10% of brain volume) at the time of transplantation have fewer grafted cells into the parenchyma [15]. Our finding was consistent with theirs. The possible reason may be due to the following two reasons: First, small infarction was associated with mild BBB deficit which led to fewer mechanically trapped MSCs. Second, recruitment forces such as chemoattractive factors released by small infarction may not enough to attract lots of MSCs whereas these chemoattractant forces and possible other factors like brain edema caused excessive cell accumulation in the large infarction group [27].

Although IA administration showed early arrival and more transplanted MSCs in the target brain, there were still some risks. New infarction developed within 24 hours in two dogs. The likely cause was microembolism caused by transplanted MSCs which in turn led to local impeded cerebral blood flow [27], [34].Besides, exogenous labeled-MSCs may cause epilepsy after transplantation which should be paid more attention in the future research.

There were some limitations of our study. First, the numbers of dogs with large and small cerebral infarction in group A were small, limiting the investigation of the relationship between initial ischemic volume and the amount of grafted cells in the brain. Second, the amount of grafted cells in the brain was analyzed by visual evaluation and T2^*^ ratio. T2^*^ value was measured by manually drawn ROIs which may lead to some bias. Third, MRI sensitivity of detecting labeled MSCs was affected by many factors, such as MRI protocol and software. Our imaging methods may not be sensitive enough for small amount of engrafted cells or tiny cell clusters. Finally, more animals were needed to confirm the reproducibility of our study.

In conclusion, it is feasible to transplant MSCs through IA route for cerebral infarction in a canine model. Successful IA administration showed diffuse distribution pattern, and large amounts of transplanted MSCs in the target brain. Both the flow status of ipsilateral MCA and infarction volume before transplantation may play an important role in the amount of grafted cells in the brain. In vivo MR imaging is useful to track SPIO-labeled MSCs for at least four weeks. However, more attention should be paid on the safety of IA approach considering the high ratio of adverse consequences.
